# Anorectal Malformation with Rectovestibular Fistula and Vaginal Agenesis: Usage of Rectovestibular Fistula as a Neovagina Followed by PSARP with Preservation of the Anal Sphincter

**DOI:** 10.1055/s-0041-1728725

**Published:** 2021-07-12

**Authors:** Antonio Dessanti, Osnel Louisma, Gabriel Steve Pierre, Nguhien Thanh Liem

**Affiliations:** 1 AOU University Hospital, University of Sassari, Sassari, Italy; 2 Department of Pediatric Surgery, Saint Damien Children Hospital, Port Au Prince, Haiti; 3 Department of Pediatric Surgery, National Children Hospital and Vinmec International Hospital, Hanoi, Vietnam

**Keywords:** anorectal malformation, vaginal agenesis, posterior sagittal anorectoplasty, vaginoplasty

## Abstract

Anorectal malformation with rectovestibular fistula associated with vaginal agenesis is rare. We report on a child in whom this combination was diagnosed at the age of 1 year. After creation of a divided descending colostomy, we chose to leave the rectum-rectovestibular fistula to function as a neovagina, while the sigmoid colon was relocated via modified posterior sagittal anorectoplasty. The colostomy was closed 6 months after the main surgery. After a follow-up of 3 years, the patient is continent for stool and urine. The introitus of the neovagina appears wide and easy to explore. We conclude that our surgical approach may be a good option for these children.

## Introduction

Anorectal malformations (ARMs) are rare congenital defects with an estimated worldwide incidence of 2 to 6 per 10,000 live births. 1 They comprise a wide range of pathologies that may involve other parts of the gastrointestinal tract as well as urinary and genital tracts. 2 3 4 5 6 7 8 9 The most common type of ARM in female patients is a rectovestibular fistula. The incidence of vaginal agenesis associated with ARM is even lower. 4 The approach to this combination was first described by Cohn and Murphy via a combined laparotomic and perineal operation. 10 The use of rectovestibular fistula as a neovagina has also been reported in combination of a posterior sagittal anorectoplasty (PSARP), 2 as described by Levitt et al. 4 We describe the case of a 1-year-old girl with ARM and rectovestibular fistula in whom we chose to leave the rectum-rectovestibular fistula to function as a neovagina, while the sigmoid colon was relocated via modified PSARP, according to Liem and Hau, and Liem and Quynh technique. 11 12

## Case Report

The previous term infant (birth weight 3.1 kg) was admitted to the Pediatric Surgery Service at Saint Damien Pediatric Hospital, Port-au-Prince, Haiti, with a diagnosis of vaginal agenesis associated to ARM with rectovestibular fistula. Until 10 months of age, the infant defecated through the rectovestibular fistula that required dilations even though it had a considerable caliber. Two months prior to clinical admission, she received a descending colostomy with distal mucous fistula. No other obvious malformations and a normal sacrum were found. Due to the lack of resources at the local hospital, no additional diagnostic imaging, for example, magnetic resonance imaging, was performed.

**Fig. 1:**
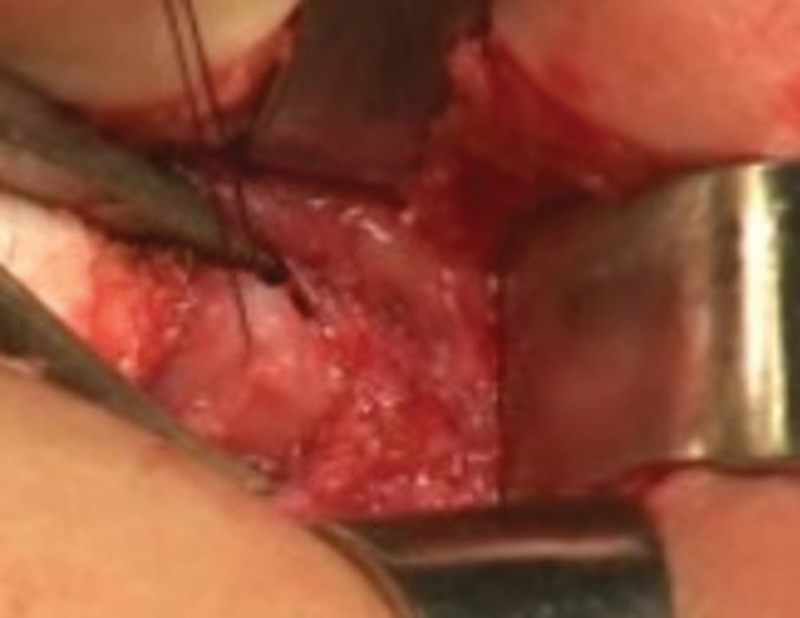
The lower part of the muscle complex was retracted down to expose the posterior wall of the rectum.

We chose to leave the rectovestibular fistula in situ to function as a neovagina and to perform a PSARP, 2 4 modified at preserving the anal sphincter as reported by Liem and Hau, and Liem and Quynh. 11 12 The patient was placed in a prone jackknife position. After locating the anal sphincter by using a neurostimulator, a posterior sagittal incision of the cutaneous and subcutaneous planes was made 1 to 2 cm over the coccyx to the superior limit area of the anal dimple. The dissection was deepened while paying attention to the midline, to avoid injuries to muscles and nerve fibers, and arrested at the level of the upper limit of the muscle complex. The muscle complex was identified and its lower part was retracted downward to expose the posterior wall of the rectum ( Fig. 1 ). The fibrotic bands between the rectal wall and the coccyx were divided and the coccyx removed aiming at gaining extra space. The rectum appeared considerably dilated to the extent that not all its circumferences could be isolated. As a consequence, it was isolated only from its lateral sides and the anterior surface of the sacrum until the peritoneal reflection which was opened in view of the subsequent laparotomy.

**Fig. 2:**
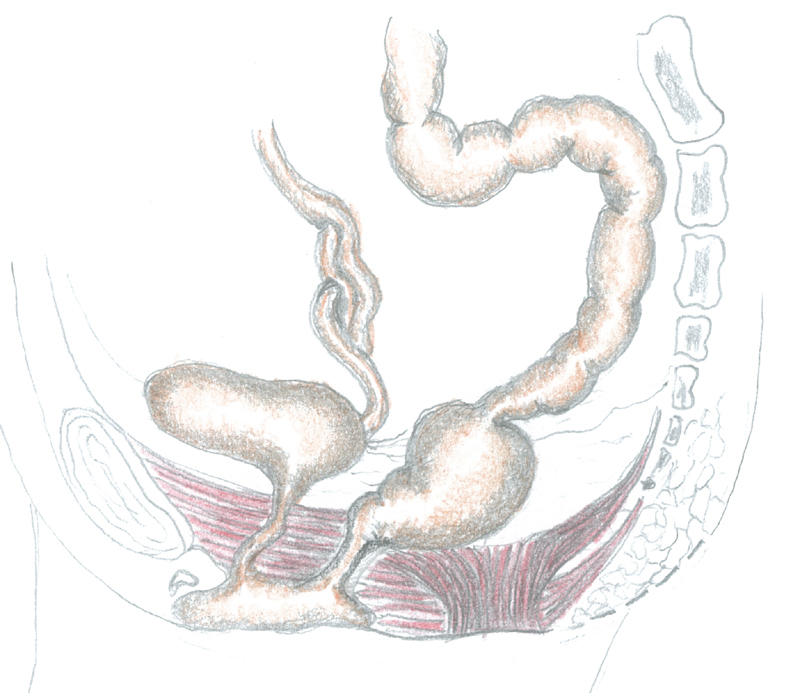
Anatomy of the patient.

After having temporarily closed the posterior sagittal skin incision to minimize the risk of bacterial contamination, the patient was placed in supine position to open the abdomen through a Pfannenstiel incision. The rectum, which was abnormally dilated until 1 to 2 cm beyond the peritoneal reflection, continued as sigmoid colon whose caliber appeared suddenly normal ( Fig. 2 ). The rectouterine Douglas' pouch was explored; while ovaries were located with fallopian tubes presenting cordlike structures, instead no structures resembling vagina and uterus were found.

**Fig. 3:**
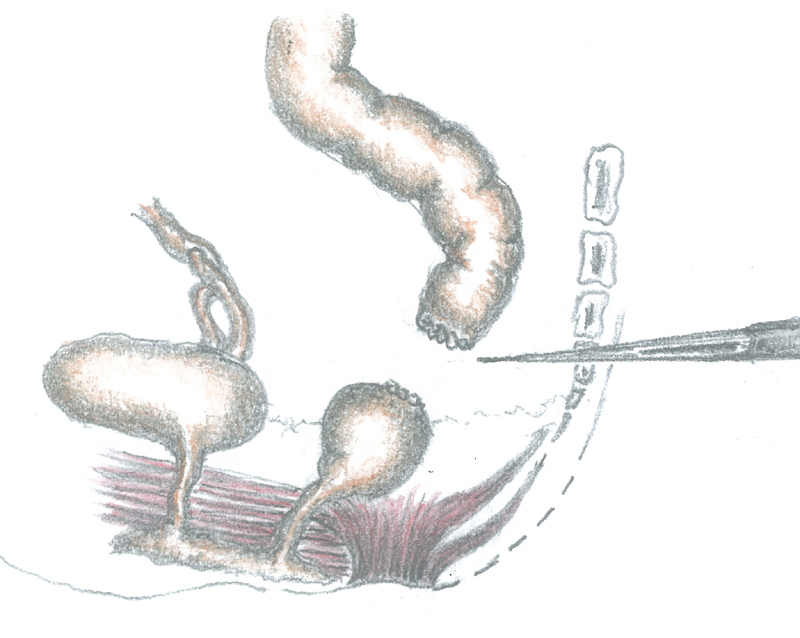
Sigmoid colon surgically separated from the dilated rectum.

The dilated rectum was surgically separated from the sigmoid colon and closed; the sigmoid colon was only temporarily sutured to avoid fecal contamination ( Fig. 3 ).

**Fig. 4:**
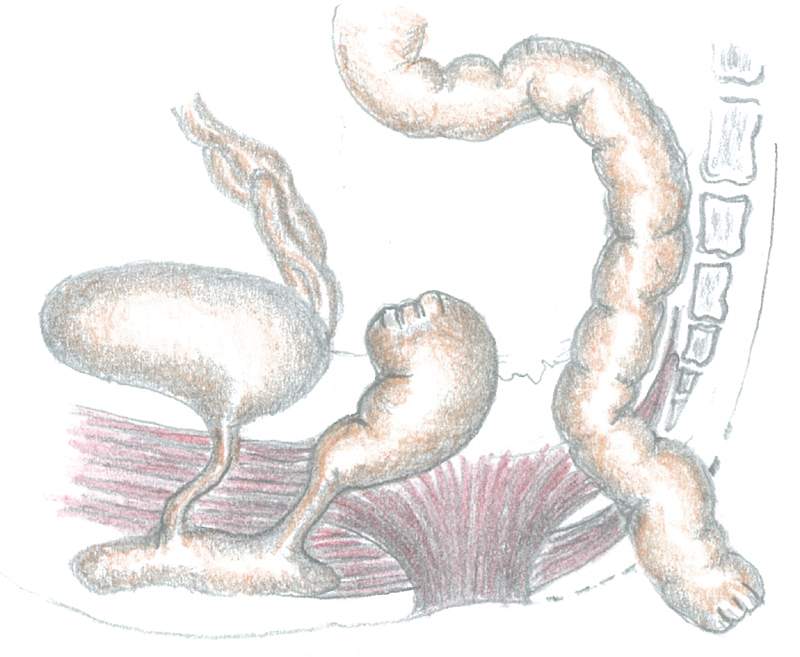
Mobilized sigmoid colon passed into the posterior sagittal space.

After binding and dissecting some branches of its vascular arcade, the sigmoid colon was then mobilized, as it needed to be easily passed without traction through the retrorectal space into the previously opened posterior sagittal space ( Fig. 4 ), which was reopened at this point.

**Fig. 5:**
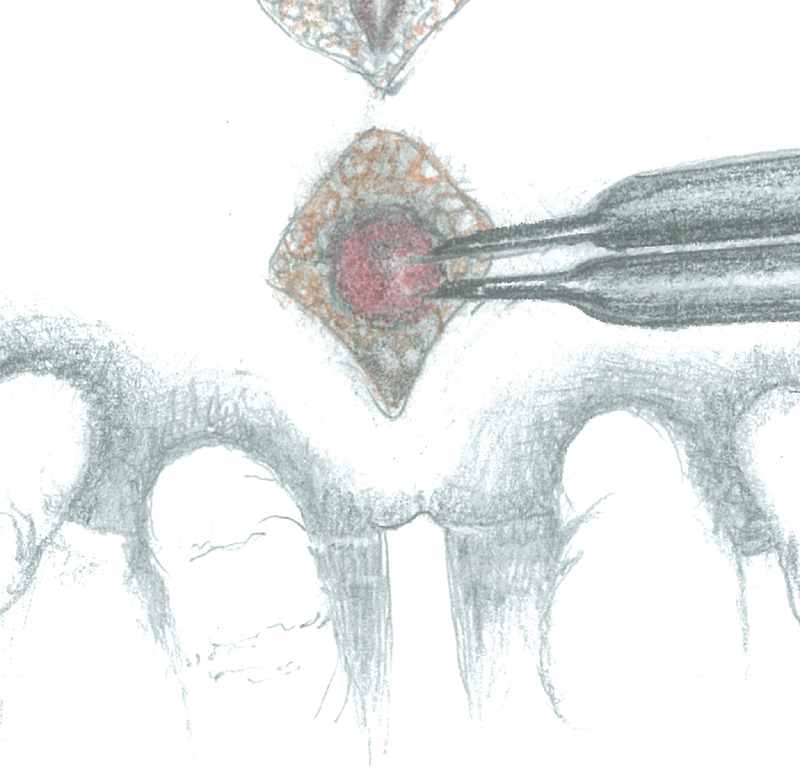
The center of the sphincter complex is defined using a neurostimulator.

**Fig. 6:**
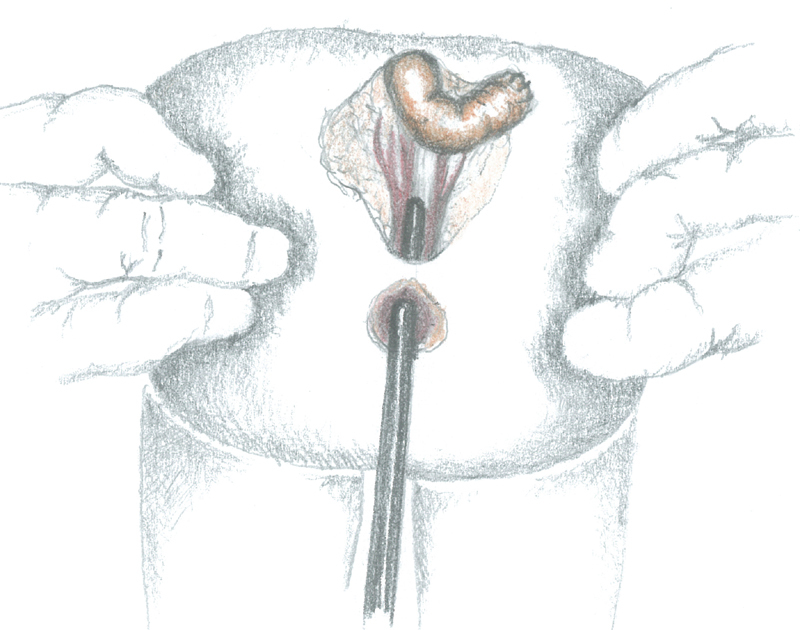
Progressive dilation of the tunnel created in the center of the sphincter complex.

Once the suprapubic incision was sutured and the patient was again in the same prone position as before, a second small inverted cutaneous Y-shape incision was made on the anal dimple, after reconfirming the location of the underlying anal muscle complex using the neurostimulator ( Fig. 5 ). Our technique was different from the original technique of PSARP described by Peña and Devries, which envisages to separate the sphincter muscle complex in the midline. 2 In contrast, we created a tunnel in the center of the sphincter muscle complex through a gentle and progressive dilation of its muscle fibers using Hegar dilators (size 6–12) ( Fig. 6 ) until reaching an acceptable diameter to enable the mobilized sigmoid colon to go through it.

**Fig. 7:**
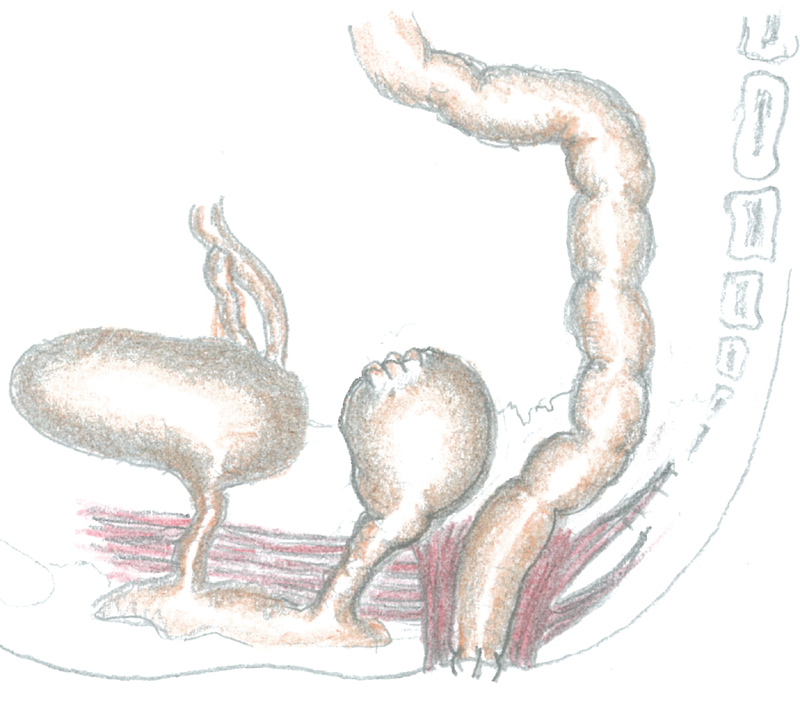
The sigmoid colon is passed through the dilated tunnel of the sphincter complex and a neoanus is created.

Consequently, the sigmoid colon, that managed to pass through the dilated tunnel of the sphincter muscle complex, was sutured to the external sphincter and to the margins of the small Y-shape cutaneous incision that had already been made in the anal dimple by using interrupted absorbable stitches ( Fig. 7 ).The posterior sagittal incision was then closed. Postoperatively, the neoanus was gradually dilated using Hegar dilators from the second week after the surgery for a total period of 1 month. The colostomy was closed after 6 months.

No complications were observed during the early postoperative period. Neither anal stenosis nor other local and general complications were observed.

**Fig. 8:**
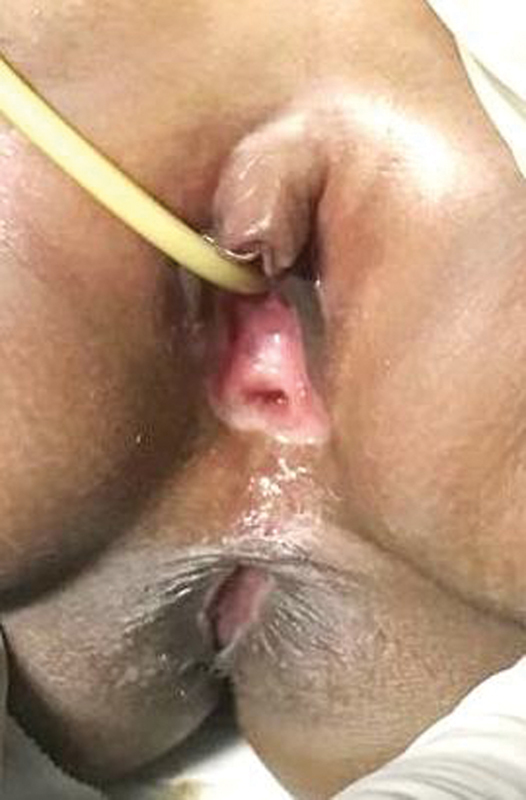
Neoanus and neovaginal introitus at 2.6 years of age.

Two years after the anoplasty and at the age of 3 years, the parents reported that a few months earlier the girl stopped using diapers as she had achieved good bowel control and always evacuated spontaneously. So, no complications occurred such as fecal incontinence (not even partially), no constipation or mucosal prolapse, nor urinary incontinence. The rectovestibular fistula, that had become the vagina introitus, appeared relatively wide and easily to explore ( Fig. 8 ).

## Discussion

Our case is a subtype of Mayer–Rokitansky–Kuster–Hauser syndrome, 6 13 caused by embryologic underdevelopment of the Mullerian duct with resultant agenesis of vagina and uterus. Vaginal agenesis associated with ARM is uncommonly seen. 4 5 6 7 9 10 13 14 15 16 17 Banu et al 18 reported 5 patients with a rectovestibular fistula and absence of vagina in their series of 563 cases.

For what concerns the reconstruction of vaginal agenesis in cases of vaginal agenesis without ARM, the first-line approach, based on safety reasons, is the primary vaginal elongation by dilations, sometimes with local estrogen, known as Vecchietti procedure 13 19 ; if no satisfactory results are achieved, during adolescence can be indicated an intra-abdominal traction applied to the perineal membrane, causing invagination over the course of a week. This technique often produces an inadequate vaginal length that might interfere with normal sexual life. Due to these reasons, other surgical options include vaginoplasty using a part of the ileus or sigmoid colon. 5 6 7 9 13 15 16

In 1956, Cohn and Murphy 10 proposed for the time to correct vaginal agenesis in ARM rectovestibular fistula type, using the rectum-rectovestibular fistula in situ as a neovagina. In other reports on vaginal agenesis, an abdominoperineal approach 14 17 and a PSARP according to Levitt et al were used. 4 20

This was also our decision: the vaginal agenesis was corrected using the rectum including the rectovestibular fistula to function as the vagina. The sigmoid colon was pulled through and relocated to be the neoanus, 2 as proposed by Levitt et al, 4 including laparotomy, but preserving the anal sphincter, according to Liem and Hau, and Liem and Quynh. 11 12

At 3 years of age follow-up, the patient showed good bowel control, which generally at that age cannot be completely achieved. 21 No clinical complications such as constipation or fecal incontinence were reported.

Although it represents a unique case, we believe that such satisfactory functional results probably also derived from the surgical technique used besides the fact that no lesions involving the sacrum were observed; unlike what takes place in the Peña and Devries' PSARP technique, based on an incision of the sphincter muscle complex in the midline, 2 the sphincter muscle complex was not divided and the sigmoid colon was pulled through a tunnel created at its center. Having left the sphincter muscle complex untouched leads us to hypothesize that the function of this important structure was preserved; this technique has already been reported. 11 12

Differently from our strategy of using the in situ rectum as a neovagina, another clinical option was to separate the rectum from urethra and relocate it within the limits of the sphincter mechanism. However, the major disadvantage of this technique is that later a more time-consuming surgery for vaginal reconstruction is required, which can also be an intervention of higher complexity. In addition, the separation of the rectum from the urethra might provoke denervation of the lower urinary tract as well as risks of denervation involving the rectum. 4

**Fig. 9:**
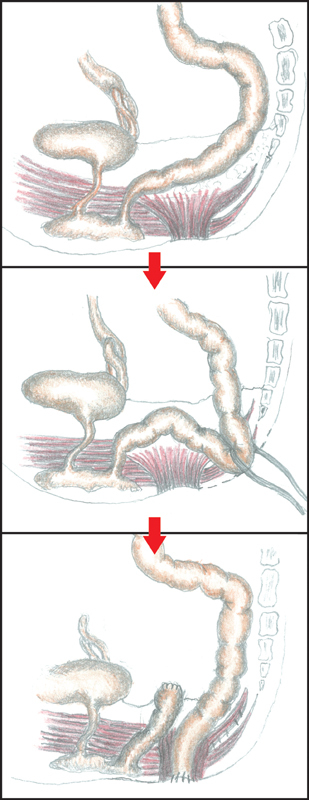
Sketch of the one-stage posterosagittal approach, preserving the muscular sphincter complex (Liem and Hau, and Liem and Quynh technique), to correct vaginal agenesis and anorectal malformation with rectovestibular fistula.

As the rectum shoved a considerable dilation, we added a suprapubic laparotomy before performing a modified PSARP with preservation of an intact anal sphincter. If the rectum had a normal caliber, we would have pulled down the proximal rectum to the neoanus ( Fig. 9 ).

The final result of our procedure was an introitus situated rather anteriorly in the vulva probably due to extra space left by the missing vagina whose space might have been taken.

We hope that the future sexual function and satisfaction will be beneficial as the terminal nerves to the vulva and rectum involved were not subjected to any surgical manipulation, unlike a lot of other surgical techniques currently used for vaginal reconstruction.

Clearly, the diameter of the new vaginal introitus will have to be assessed during puberty; in the case of insufficient diameter, an event highly expected to occur, it might be widened using the wide walls of the rectovestibular fistula.

Also, at long-term follow-up, the sensitivity of the new vaginal introitus will have to be assessed.

## Conclusion

Our surgical strategy based on the decision to leave the rectum in situ with its rectovestibular fistula used as a neovagina before creating a neoanus via a PSARP may provide an effective alternative to other vaginal reconstruction strategies using more complex reconstruction techniques.

## Notes

New Insights and the Importance for the Pediatric Surgeon ARM is one of the most complex congenital anomalies involving several organs. The association of ARM with vaginal genesis, as it is described in our case, is a very rare and complex event to be corrected surgically.

## References

[1] de Blaauw I Wijers C HW Schmiedeke E First results of a European multi-center registry of patients with anorectal malformations J Pediatr Surg 2013 48 12 2530 2535 24314198 10.1016/j.jpedsurg.2013.07.022

[2] Peña A Devries P A Posterior sagittal anorectoplasty: important technical considerations and new applications J Pediatr Surg 2020 30 05 447 451 10.1016/s0022-3468(82)80448-x 6761417

[3] Banu T Karim A Adel M G Multicenter study of 342 anorectal malformation patients: age, gender, Krickenbeck subtypes, and associated anomalies Eur J Pediatr Surg 2020 30 447 451 31655491 10.1055/s-0039-1695789

[4] Levitt M A Stein D M Peña A Rectovestibular fistula with absent vagina: a unique anorectal malformation J Pediatr Surg 1998 33 07 986 989, discussion 990 9694082 10.1016/s0022-3468(98)90519-x

[5] Wesley J R Coran A G Intestinal vaginoplasty for congenital absence of the vagina J Pediatr Surg 1992 27 07 885 889 1640339 10.1016/0022-3468(92)90392-k

[6] Wester T Tovar J A Rintala R J Vaginal agenesis or distal vaginal atresia associated with anorectal malformations J Pediatr Surg 2012 47 571 576 22424355 10.1016/j.jpedsurg.2011.09.040

[7] Pandya K A Koga H Okawada M Coran A G Yamataka A Teitelbaum D H Vaginal anomalies and atresia associated with imperforate anus: diagnosis and surgical management J Pediatr Surg 2015 50 03 431 437 25746703 10.1016/j.jpedsurg.2014.07.010

[8] Vilanova-Sanchez A Reck C A McCracken K A Gynecologic anatomic abnormalities following anorectal malformations repair J Pediatr Surg 2018 53 04 698 703 28797517 10.1016/j.jpedsurg.2017.07.012

[9] Skerritt C Vilanova Sánchez A Lane V A Menstrual, sexual, and obstetrical outcomes after vaginal replacement for vaginal atresia associated with anorectal malformation Eur J Pediatr Surg 2017 27 06 495 502 27846665 10.1055/s-0036-1593610

[10] Cohn B D Murphy D R Imperforate anus with agenesis of the vagina Ann Surg 1956 143 03 430 432 13303077 10.1097/00000658-195603000-00017 PMC1465123

[11] Liem N T Hau B D Long-term follow-up results of the treatment of high and intermediate anorectal malformations using a modified technique of posterior sagittal anorectoplasty Eur J Pediatr Surg 2001 11 04 242 245 11558014 10.1055/s-2001-17155

[12] Liem N T Quynh T A One stage operation through modified posterior sagittal approach preserving the sphincter intact for anal agenesis with rectovestibular fistula J Pediatr Surg 2015 50 04 634 637 25840077 10.1016/j.jpedsurg.2015.01.003

[13] Committee on Adolescent Health Care Mullerian agenesis: diagnosis, management and treatment Obstet Gynecol 2018 131 01 e35 e42 29266078 10.1097/AOG.0000000000002458

[14] Ein S H Stephens C A Vaginal construction in children with absent vagina and imperforate anus J Pediatr Surg 1971 6 04 435 439 5563883 10.1016/s0022-3468(71)80004-0

[15] De la Torre L Cogley K Calisto J L Santos K Ruiz A Zornoza M Vaginal agenesis and rectovestibular fistula. Experience utilizing distal ileum for the vaginal replacement in these patients, preserving the natural fecal reservoir J Pediatr Surg 2016 51 11 1871 1876 27567309 10.1016/j.jpedsurg.2016.08.003

[16] Günşar C Genç A Sencan A Dağlar Z Alparslan O Mir E MURCS association and rectovestibular fistula: case report of a patient treated with one-stage posterior sagittal anorectoplasty and sigmoid loop vaginoplasty J Pediatr Surg 2003 38 02 262 264 12596120 10.1053/jpsu.2003.50060

[17] Fujiwara Y Oizumi T Sasahara M Kato E Kakizaki G A case of congenital imperforate anus and absent vagina with a functioning uterus Tohoku J Exp Med 1974 113 03 283 289 4454061 10.1620/tjem.113.283

[18] Banu T Hannan M J Aziz M A Hoque M Laila K Rectovestibular fistula with vaginal malformations Pediatr Surg Int 2006 22 03 263 266 16307276 10.1007/s00383-005-1594-3

[19] Vecchietti G Neovagina nella sindrome di Rokitansky-Küster-Hauser Attual Ostet Ginecol 1965 11 02 131 147 5319813

[20] Levitt M A Bischoff A Breech L Peña A Rectovestibular fistula--rarely recognized associated gynecologic anomalies J Pediatr Surg 2009 44 06 1261 1267, discussion 1267 19524751 10.1016/j.jpedsurg.2009.02.046

[21] Issenman R M Filmer R B Gorski P A A review of bowel and bladder control development in children: how gastrointestinal and urologic conditions relate to problems in toilet training Pediatrics 1999 103(6 Pt 2):1346 1352 10353952

